# Understanding leaders’ learning pathways in inclusive leadership: integrating learning logs with guided reflection

**DOI:** 10.1080/13678868.2025.2505836

**Published:** 2025-05-19

**Authors:** Rachel Verheijen-Tiemstra, Anje Ros, Marc Vermeulen, Rob F. Poell

**Affiliations:** a School for Child Studies and Education, Fontys University of Applied Sciences, Eindhoven, The Netherlands; b School of Social and Behavioral Sciences, Tilburg University, Tilburg, The Netherlands; c TIAS School for Business and Society, Tilburg University, Tilburg, The Netherlands

**Keywords:** Human Resource Development, leadership development, inclusive leadership, experience-based workplace learning, qualitative research

## Abstract

In the field of Human Resource Development (HRD), inclusive leadership is emerging as an innovative approach to leadership development, reflecting the growing importance of collaboration in the evolving work landscape. This study examines the development of inclusive leadership in an interprofessional education context. A professional development programme consisting of four sessions was conducted, where participants, as part of the programme, kept learning logs and reflective interviews with each participant were held between the sessions. The research question was as follows: How do school and childcare leaders develop inclusive leadership behaviours in the workplace during a dedicated Professional Development Programme? Analysis of the learning logs revealed that participants primarily engaged in a learning pathway of enactment, indicating direct experimentation in their professional context. The reflective interviews uncovered an extended repertoire of learning pathways that frequently incorporated learning through reflection. Our study shows the effectiveness of integrating learning logs with guided reflection as tools to assess and further encourage professional development. On a theoretical level, the Interconnected Model of Professional Growth (IMPG), which we used for mapping learning pathways, enabled us to effectively analyse learning pathways in attaining perceived professional growth in inclusive leadership

## Introduction

Recently, inclusive leadership has garnered increased research attention (Roberson & Perry, 2021; Veli Korkmaz et al., 2022). Inclusive leadership is defined as a leadership approach that fosters positive outcomes by creating an inclusive organisational climate that facilitates the incorporation of a broad range of viewpoints and concepts, consequently augmenting decision-making procedures and elevating the effectiveness of diverse teams (Ashikali et al., 2021). Inclusive leadership advocates diversity, equity, and inclusion through the active involvement and engagement of team members, encouragement of their contributions, and recognition of their distinctive perspectives and strengths (Randel et al., 2018). Byrd (2022a) asserted the importance of inclusive leadership in the field of Human Resource Development (HRD) as a means of exploring innovative leadership development approaches that are adaptive to the evolving work landscape. In contexts where team members experience strong fault lines, inclusive leadership behaviour (ILB) emerges as a suitable leadership approach because it has a positive impact on employee behaviour, reduces turnover (Nishii & Mayer, 2009), and enhances employees’ perceived insider status (Naseer et al., 2023; Shah et al., 2022).

A context where such strong fault lines are perceived is the context of child centres, an innovative provision where primary education is combined with out-of-school care, pre-school (ages 2–4), and day care (ages 0–4) (Fukkink & Lalihatu, 2020). Recent research by Verheijen-Tiemstra et al. (2024a) illuminated the need for enhanced inclusive leadership among school and childcare leaders. Their study revealed a focus on fostering belongingness rather than valuing uniqueness, while both aspects are crucial for inclusion. In addition, it identified leaders’ displays of both positive and counterproductive behaviours, hence hindering the full development of an inclusive organisational climate.

Building upon the aforementioned, this study was devised to establish and simultaneously study a professional development programme (PDP). The primary objective of this programme was to facilitate participants’ advancement in their inclusive leadership behaviour, with the overarching goal of promoting interprofessional collaboration (IPC) between employees of both schools and childcare. The significance of this study is heightened by the crucial role that school leaders’ workplace learning plays in their professional development and effectiveness. However, there is a limited understanding of the informal learning processes that underpin how school leaders acquire knowledge (Ringling et al., 2021). Within the education ecosystem, research on the professional development of childcare leaders is perhaps even more scarce (Gibbs, 2021; Grantham-Caston & DiCarlo, 2023). Nevertheless, Coleman et al. (2016) stated that a key characteristic of high-performing childcare leaders is their emphasis on learning as a means of improving quality, including engaging in self-reflection. Hulsbos et al. (2015) earlier emphasised the value that school leaders place on informal workplace learning through improvement and reflection. Ringling et al. (2021) stated that school leaders’ informal learning opportunities are just as important as formal ones, and research on school leaders’ workplace learning can be extremely beneficial to the field of leadership development. To narrow this gap, conducting research that integrates formal learning opportunities while simultaneously tracking participants’ informal workplace learning experiences is essential. This approach aims to provide insights into how these leaders learn and to understand how the learning processes between formal learning activities and informal workplace learning can mutually reinforce each other. Thus, the following research question arises that we aim to address in this paper: *How do school and childcare leaders develop their inclusive leadership behaviours in the workplace during a dedicated Professional Development Programme*?

With this paper, we aim to achieve several goals. First, we aim to expand the body of knowledge on inclusive leadership by providing insights into how leaders develop their inclusive leadership behaviours. Inclusive leadership is a novel approach to leadership that has not been widely adopted in human resource development (HRD) literature Byrd (2022b). However, both practitioners and researchers have embraced inclusive leadership as a strategy with the potential to enhance task performance while simultaneously ensuring work engagement and well-being of employees (Gürbüz et al., 2024; Veli Korkmaz et al., 2022). Moreover, Boutwell and Smith (2023) identified a significant gap in the literature regarding the role of inclusive leaders in fostering organisational practices aimed at strengthening interpersonal relationships while simultaneously driving organisational transformation. This study seeks to address this gap by exploring how leaders themselves develop inclusive leadership behaviours in the context of transformation, as manifested in the emergence of child centres for education and care in the current research. Furthermore, we aim to enhance the existing knowledge base on the learning processes of school and childcare leaders, a field that has been relatively under-researched (Daniels et al., 2023). Thus, the study also contributes to the ongoing debate in the field of HRD regarding professional learning and culture. As leadership behaviour is always developed within the context of a particular national culture, it is shaped by the collective expectations of members within an organisation or society, which are informed by shared values, beliefs, and norms (Kortsch et al., 2023). This paper contributes to the body of knowledge regarding the development and implementation of inclusive leadership in the Dutch context, which is typically characterised by a national culture with low power distance.

## Literature review

In this section, the content of the PDP, specifically inclusive leadership, will be discussed, along with a review of school leaders’ learning and the Interconnected Model of Professional Growth (IMPG), which serves as a model for mapping learning pathways.

### Content of the PDP: inclusive leadership

Interprofessional teams consist of individuals with diverse educational backgrounds and expertise who collaborate towards specific goals (Bryson et al., 2015). While this diversity can enhance outcomes through the integration of various perspectives, it can also lead to conflicts (Dwertmann et al., 2016; Mitchell et al., 2015; Shemla & Wegge, 2019). Based on this, establishing an inclusive organisational climate is advocated (Ashikali et al., 2021; Boekhorst, 2015; Shemla & Wegge, 2019), where individuals feel they are valued members of the workplace due to policies, practices and procedures that satisfy their needs for belongingness and uniqueness, without being pressured to assimilate (Shore et al., 2011). Leaders play a crucial role in creating a strongly inclusive organisational climate by prioritising this in their day-to-day work (Ostroff & Bowen, 2016), using both direct and indirect mechanisms, such as encouraging or discouraging actions (E. L. Perry et al., 2021) and influencing team members’ perceptions through their own behaviours (Boekhorst, 2015).

From the perspective that inclusive leadership facilitates the efficient operation of diverse work groups, Randel et al. (2018) developed a theoretical model of inclusive leadership comprising two dimensions that facilitate inclusion in groups: facilitating belongingness and valuing uniqueness. This model builds upon the earlier work of Shore et al. (2011) who argued that uniqueness and belongingness work together to create inclusion. To achieve inclusion, both belongingness and uniqueness must be addressed. However, leaders may prioritise belongingness, as they are used to emphasising collective goals over valuing uniqueness. This is confirmed by Verheijen-Tiemstra et al. (2024a), who revealed that leaders do not always apply inclusive leadership in practice, with behaviours related to uniqueness being particularly infrequent. In their study, they operationalised the ILB framework in the context of IPC in child centres. Table 1 provides an overview of the two dimensions of ILB, their main aspects, and their definitions.

**Table 1.** Framework for inclusive leadership in IPC: aspects and definitions, building on the inclusive leadership framework by Randel et al. (2018).

Dimensions	Aspects and definition
1. Facilitating belongingness	A. **Supports individuals as group members**: strengthens team cohesion and creates a sense of community through which school and childcare staff feel connected and feel pride in being members of the child centre team.
	B. **Ensures justice and equity**: makes sure that all employees from both sectors, independent of their (professional) background, are treated with equal respect by distributing *and* recognising justice and equity.
	C. **Organises participation and information sharing**: organises joint responsibility and decision-making processes based on knowledge *and* information sharing.
	D. **Is a role model for belongingness**: is a true role model by setting high standards for themselves through the establishment of robust and stable relationships with both childcare and school staff.
2. Indicating value of uniqueness	A. **Encourages diverse contributions**: supports perspectives and orientations from staff from both sectors that may contribute to performance, even when those perspectives differ from mainstream views, by creating an environment that acknowledges, welcomes, and accepts different approaches, styles, perspectives, and experiences.
	B. **Helps staff to fully contribute**: makes sure that group members do not hold back or encounter obstacles in contributing to the group.
	C: **Is a role model for valuing uniqueness**: shows high standards for themselves in their own approach to employees from the other sector that shows appreciation for the professional uniqueness of these employees.

It is noteworthy that effective inclusive leadership, rooted in relational leadership theories, surpasses mere behavioural expectations, as mentioned in Table 1, necessitating a profound shift in perspective (Boutwell & Smith, 2023). To provide employees with experiences that reflect inclusion, leaders must adopt a mindset that goes beyond conventional notions of leadership as a social influence process (Nishii & Leroy, 2022; Roberson & Perry, 2021). In their view, this requires signal sensitivity and a profound capacity to discern and respond to the diversity dynamics inherent in relational processes.

Nishii and Leroy (2022) advocated a broader perspective that encompasses not only social inclusion but also informational and task-related inclusion, which is particularly salient within IPC contexts. Inclusive leaders should therefore actively counteract the detrimental influence of stereotype-based status dynamics, which could oppress team members with a lower (professional) status to conform to the dominant majority. Building on relational-cultural theory, Boutwell and Smith (2023) further illustrated that failures in inclusivity often originate from power imbalances, where individuals in dominant positions may unintentionally suppress the voices of others.

In conclusion, we propose that a comprehensive PDP should extend its focus beyond cultivating behaviours associated with inclusive leadership. It should also devote attention to nurturing a mindset conducive to embracing inclusive leadership. This multifaceted approach underscores the significance of fostering self-reflection on one’s own actions, a process that can be undertaken by the learner individually, as well as through collaborative learning with peers and one-on-one reflective interviews.

### School leaders’ learning and the interconnected model of professional growth

Although research on the professional development of school leaders is somewhat limited, and research on the professional development of childcare managers is scarce, various insights can be gleaned from the literature. The comprehensive reviews by Daniels et al. (2019) and Hulsbos et al. (2015) underscore the significance of context-specific, collaborative, and reflective learning. Self-directed reflective learning is highly valued by school leaders, but it is also perceived as challenging (Neeleman, 2019).

Recent research by Greenan (2023) underscores the challenge of immediate reflection, mentioning the perceived time constraints caused by the prioritisation of performance over reflection (Nesbit, 2012). Greenan (2023) therefore recommends supported reflection as a means of promoting deep reflection. In the same vein, Nakamura et al. (2022) advocated incorporating guided reflection approaches to enhance leadership development, based on their research using neuroscience methods that compared neurocognitive mechanisms during individual and guided reflection.

### The interconnected model of professional growth

The aforementioned insights indicate that when studying PDPs, it is imperative to attain an understanding of the combination of learners’ perspectives and behaviours, alongside an analysis of the learning pathways they have undertaken. The Interconnected Model of Professional Growth (IMPG), as proposed by Clarke and Hollingsworth (2002), may be deemed a pertinent analytical framework to scrutinise the learning pathways of school leaders, stemming from workplace learning and with a strong focus on reflection-based learning.

**Figure 1.**
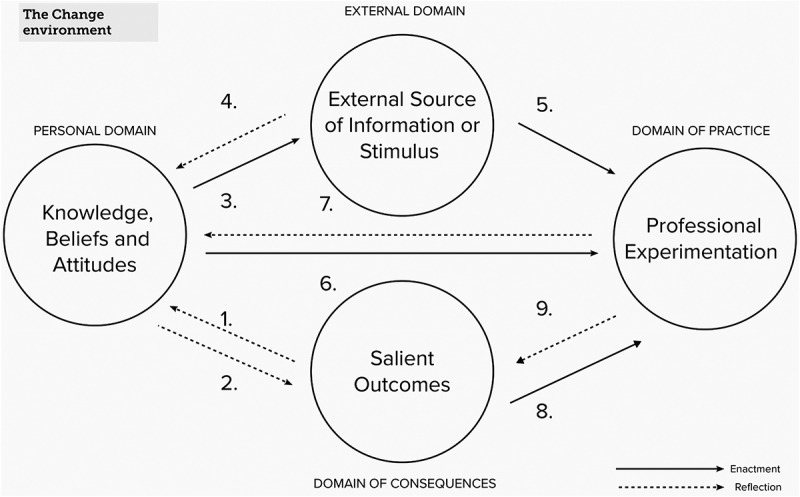
The interconnected model of professional growth (Clarke & Hollingsworth, 2002, p. 957), showing the 9 learning pathways.

The model shown in Figure 1 presents an approach to professional learning that is valuable for theorising, understanding and enhancing teacher professional development and professional growth (Boylan, 2021). The model’s concept encompasses four domains where learning can take place: the personal domain (knowledge, beliefs and attitudes), the domain of practice (professional experimentation), the domain of consequence (salient outcomes), and the external domain (sources of information, stimulus or support) (Clarke & Hollingsworth, 2002, p. 950).

Changes in these four domains can be identified through two processes of enactment and reflection, comprising three different learning pathways: a direct pathway from the personal domain to the domain of practice and two indirect pathways from the personal domain via the external domain and the domain of consequences. Learning pathways comprise ‘two or more domains together, with the reflective or enactive links connecting these domains’ (Clarke & Hollingsworth, 2002, p. 958). According to these authors, not all changes are considered ‘professional growth’, as brief changes may be quickly abandoned. According to Justi and van Driel (2006), change can be considered ‘professional growth’ when more than two domains are involved or when a particular change is sustained over an extended period. Thus, the model surpasses other learning pathway models, such as those proposed by Guskey and Desimone (Boylan et al., 2018), in that it proposes an iterative approach that considers diverse dynamics that allow for multiple growth pathways between domains. This approach is based on the belief that learning is a continuous and complex process (E. Perry & Boylan, 2018). The IMPG is extensively cited not only in empirical research on teacher learning in various fields, such as mathematics and Lesson Study (Schipper et al., 2017), but also in research among science museum educators as learners (Piqueras & Achiam, 2019), indicating the model’s wide applicability. This makes it a promising model for studying the learning processes of school and childcare leaders.

### Conceptual framework

**Figure 2.**
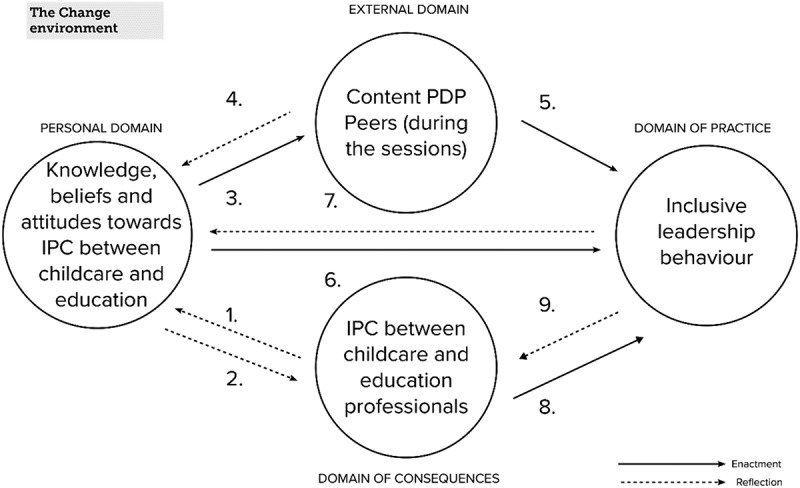
Conceptual framework learning pathways in inclusive leadership.

Based on the preceding literature review, we propose the following conceptual framework for learning pathways in inclusive leadership, as depicted in Figure 2. This framework will serve as the foundation for the empirical study.

Figure 2 illustrates how we operationalised the four domains of the IMPG to develop inclusive leadership as outlined in the literature review. The *personal domain* encompasses knowledge, beliefs, and attitudes on the facilitation of IPC between childcare and education. The *external domain* refers to the sources that provide knowledge or practices, consisting of the PDP’s content and learning from and with peers during sessions regarding ILB to enhance IPC. The *domain of practice* refers to situations in which participants showed intentional action using their knowledge of ILB during the PDP. The *domain of consequences* refers to the domain where outcomes (here: increased IPC as a consequence of ILB) are interpreted, while also including signals that would indicate the absence of IPC.

## Methodology

Our study follows a qualitative longitudinal design, as longitudinal studies are especially well suited for capturing processes that unfold over a specified duration (Anderson, 2017; Ritchie et al., 2014), such as learning processes during a PDP. In this context, the purpose is to clarify various facets of transformation (in this case, professional growth in ILB) and illuminate the mechanisms through which they manifest. Brown et al. (2011) underscored the significance of qualitative research on PDPs because of the rich insights it offered compared with conventional quantitative methods that rely on closed-ended questions. Within this qualitative research, the first author assumed a tripartite role, acting in the capacities of researcher, educator, and intervention leader. To prevent any undue bias in the data analysis because of this positionality, each stage of the research process involved extensive collaboration and deliberation among the entire author team. In doing so, we drew on insights from Consensual Qualitative Research (Hill et al., 2005), a qualitative approach that incorporates elements of phenomenological approaches but combines them with discussion and reflection between researchers to achieve consensus and avoid potential biases. In this approach, there is a mutually beneficial dynamic. The researcher collected data while facilitating the learning process through the probes used to help the participant explore their experiences. Adoption of this approach included the use of semi-structured interview protocols, which comprised a set of scripted questions and a list of suggested probes to help participants explore their experiences in more depth, and data analysis based on the use of categories rather than reporting frequencies or percentages.

### Trustworthiness and credibility

To ensure trustworthiness and credibility in this qualitative research, we followed several procedures. In terms of data collection, the learning logs and reflective interviews as multiple data sources also played a role in ensuring trustworthiness and credibility, as the reflective interviews allowed for validation of what was written in the learning logs. Furthermore, member check procedures, as suggested by Hill et al. (2005), ethical approval, and thick descriptions through the presentation of rich, direct quotations were also employed, as suggested by Anderson (2017). In addition, following Anderson (2017), a conceptual framework grounded in the literature was used to avoid the charge of being informed only by pragmatic considerations.

### Professional development programme

**Figure 3.**
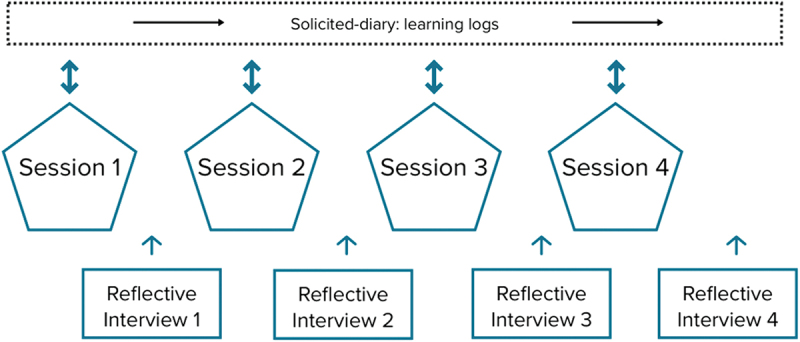
Overview of data collection.

As shown in Figure 3, the PDP comprised four half-day sessions spanning October 2021 to March 2022, with interspersed reflective interviews serving both as a data collection method and as an intervention. Ethical approval was granted by the Institutional Ethical Board on 25 May 2021, and informed consent was obtained from all participants. Participants were briefed on study objectives and data collection procedures. The sessions, led by the first author, facilitated discussions on child centre development, IPC, and inclusive leadership. Six design criteria, derived from three comprehensive studies on leadership development (Daniels et al., 2019; Fluckiger et al., 2014; Lacerenza et al., 2017), were identified, encompassing aspects related to the preparation and execution of the PDP. These design criteria were: voluntary participation, conducting a needs analysis prior to the programme, use of multiple methods (evidence-informed, theoretically-grounded and practice-oriented), fostering collective participation and collective learning, time-rich (spread over multiple sessions), and promoting self-regulated learning, based on participants’ own learning experiences and reflection on those experiences (Verheijen-Tiemstra et al., 2024b). An elaborate script aligned with these design criteria and session objectives, was meticulously prepared for each meeting.

### Positionality of place

The participants in the PDP and data collection were school leaders, responsible for primary education, and leaders responsible for full-day care, Early Childhood and Education (ECEC) and out-of-school (OSC) care (hereafter childcare leaders). They work together at child centres in the Netherlands, while being appointed from their own sector. It is worth mentioning that the childcare sector in the Netherlands has long been driven by market forces, with the aim of influencing the employment of mothers with young children by providing childcare as a commercial service (Portegijs et al., 2018). However, the reality is more complex, as privatisation has not entirely superseded the sector’s public value, as evidenced by the provision of ECEC for children with a (risk of) educational disadvantages. When the positionality of place from a cultural values perspective is considered, the Netherlands can be classified within the Scandinavian or Nordic cultural zone (Ronen & Shenkar, 2013), a cluster characterised by a low power distance, with a tendency towards egalitarian views on the leader–employee relationship (Soerensen et al., 2017). However, Den Hartog and De Hoogh (2024) identified a discrepancy between cultural values (how things should be) and cultural practices (how things are). This can also be an issue in the education sector, where ‘respect, status and expertise’ are powerful professional norms (Edmondson et al., 2016). An example of this is the dissonance expressed by school leader Daphne, who recently started as a school leader and who, through participation in the PDP, has become more aware of the apparent distance between how things should be and what she is actually aware of:

I am much more aware of my position as a school leader now. I never experienced a school leader as someone who is above me [when she was not a school leader herself], and I never felt that I had to change my behaviour because of something the school leader said. Furthermore, I do notice now that there is a culture here of ‘if the school leader says it, you must do it’. As a result, I must pay much more attention to when and how I say things because I apparently may cause that reaction.

(Daphne, school leader)

### Recruitment of participants

Participants in the PDP and data collection were school and childcare leaders who collaborated with eight child centres in the Netherlands. Considering that voluntary participation is an effective criterion to achieve learning and participation from various organisations was intended, recruitment was organised using convenience sampling through a call in a newsletter. Pairs of leaders were invited to participate in a study on leadership in child centres and were given the inclusion criteria for participation. The leaders of the 10 child centres showed interest and received further information on the purpose and approach and the conditions for participation. In total, 17 participants from eight child centres met the inclusion criteria and engaged in the entire PDP: seven child centres with two leaders responsible and one child centre where responsibility was divided among three leaders.

### Data collection

Considering the qualitative longitudinal design of our study and pursuing triangulation of data sources and perspectives, we draw on learning logs written by the participants in their solicited diary and reflective interviews that occurred after each session.

#### Research-solicited diary: learning logs

This method is well suited for research that extends over a certain period, research that generates real-time data (compared to an interview), and research that may involve sensitive topics (Milligan & Bartlett, 2019). Participants received a semi-open digital reflection diary and were asked to describe, using prompts that helped structure their thoughts, a situation they considered a powerful learning experience. Powerful Learning Experiences (PLEs) are defined as adult learning opportunities that expand and shift leadership perspectives, thus having the potential to enhance leadership capabilities and facilitate profound engagement in leadership roles (Cunningham et al., 2019). In this semi-open format, respondents briefly outlined the PLE and described their actions in that context using keywords. In total, 17 participants completed 107 learning logs.

#### Online reflective interviews

A reflective interview is a fundamental and appropriate method in addition to a solicited diary (Filep et al., 2018) from a rigour perspective because it allows participants to interpret their notes. Therefore, in between the sessions, brief online interviews via MS Teams were conducted with each participant, asking reflection questions based on a powerful learning experience from the learning log diary that was selected by the participant as their most powerful learning experience, such as ‘What makes this meaningful?’ When it became clear during a reflective interview that a participant had not completed any learning logs, the participant was invited to describe a relevant learning experience on the spot, and the conversation proceeded from there.

### Data analysis

To code and analyse the data, the software programme Atlas.ti 23 was used for both the learning logs and reflective interviews. In our analysis, we systematically coded for aspects of ILB and learning pathways, as we will elaborate below. To this end, we adopted the procedural steps Justi and van Driel (2006) used in their study, employing the IMPG. Following Hill et al. (2005), each step was discussed within the author team until a consensus was reached on the content and implementation of each step.

The initial step, operationalisation of the domains, is outlined in the conceptual framework that serves as the basis for the empirical study.

In step 2, we operationalised the learning pathways in the context of our study and numbered each learning pathway. Table 2 presents a description of each learning pathway based on the criteria Justi and van Driel (2006) used as a guide in analysing the data, but adjusted for the content of our PDP instead. In accordance with Clarke and Hollingsworth (2002), we also coded for multiple learning pathways, which consist of a chain of pathways, thus connecting more than two domains.

**Table 2.** Criteria for establishment of learning pathways in the four domains.

Learning pathway	Description, based on Justi and van Driel (2006) and adjusted for our study
1. CD-PD [from consequences to personal]	When the learner reflects on results of professional action (e.g. exercising ILB), as evidenced by signals such as verbal or nonverbal feedback from employees, and translates that into practical knowledge, assumptions, beliefs or attitudes for the future.
2. PD-CD [from personal to consequences]	When a specific aspect of the content knowledge, beliefs and attitudes around IPC, helps the learner reflect on a specific result as a consequence of their action.
3. PD-ED [from personal to external]	When a specific aspect of the original knowledge/beliefs/attitudes/influences what the learner has said or done during one of the learning activities.
4. ED-PD [from external to personal]	When input from sources of the PDP (knowledge and/or peers) leads to expansion/change of own knowledge, beliefs, attitudes or assumptions.
5. ED-DP [from external to practice]	When input sources from the PDP (knowledge and/or peers) lead to the learner behaving differently in the professional practice, for example, by performing different and/or new activities that they carried out after the meeting.
6. PD-DP [from personal to practice]	When the learner shows intentional ILB after the sessions, based on practical knowledge, beliefs or attitudes.
7. DP-PD [from practice to personal]	When something the learner did in the professional practice leads to reflection, where their own assumptions, practical knowledge, beliefs or attitudes change.
8. CD-DP [from consequences to practice]	When the learner reflects on a specific result (e.g. employees showing more IPC) and this changes their own practical knowledge/assumptions/attitudes/opinions.
9. DP-CD [from practice to consequences]	When something that participants did in their ILB practices, caused specific outcomes that they recognise.

In step 3, the first and second authors coded text fragments from the first five interviews based on the conceptual framework. This was done in two waves, in which differences and similarities were discussed and presented to the entire author team after each wave. This resulted in minor adjustments in the external domain regarding collaboration between leaders from the same child centre. When this type of collaboration occurred during the sessions, it was decided to code it in the external domain. After the second wave, a high level of agreement was achieved, and the remaining interviews were coded by the first author.

In step 4, we applied the multiple-label classification framework proposed by Hill et al. (2005). To avoid undue emphasis on frequencies or percentages, this model uses label categories to characterise occurrence. The categories used in this classification to describe the frequencies of cases are ‘general’, ‘typical’, ‘variant’, and ‘rare’. To prevent potential connotations, we replaced the term ‘variant’ with ‘occasional’. Given that the classification framework was derived from insights gathered from a lower number of cases, we determined the number of participants who described a learning pathway within each category. This approach allowed us to meaningfully classify our sample’s learning pathways. Consequently, we categorised learning pathways as ‘general’ if mentioned by 16 or 17 participants, ‘typical’ if mentioned by 8 to 15 participants, ‘occasional’ for 3 to 7 participants, and ‘rare’ for behaviours mentioned by only one or two participants. To identify multiple learning pathways, we subsequently conducted a code-occurrence analysis using the AND operator in Atlas.ti on the fragments coded with a learning pathway, followed by a code-document table based on binary data to identify patterns of common (combinations of) learning pathways and prevalent inclusive leadership behaviours. These analyses were subsequently reviewed and discussed by the authors before being presented in the finding section.

#### Learning logs

The analysis of the learning logs proceeded as follows. Each learning log was coded for both the dimension of ILB (for example, ‘facilitating belongingness’, see Table 1), and the pursued learning pathway, based on the brief description of the learning experience provided by the participant (see Table 2). If the relevant aspect (for example, ‘strengthening interpersonal relationships’) was also described, this subcategory was also coded using the descriptions in Table 1.

While participants were encouraged to complete a learning log on a weekly basis or at least once between the sessions, this was not mandatory and resulted in a notable disparity in the number of learning logs submitted. In total, 110 coded fragments were related to inclusive leadership behaviour and 144 coded fragments were related to the IMPG analysis model. This means that multiple learning pathways were addressed in some learning logs.

#### Reflective interviews

Not all participants could participate in the originally planned four interviews because of illness or other circumstances beyond their control. In total, 59 (of 68) reflective interviews were conducted. Again, it was first coded by dimension of ILB, followed by the learning pathway. The data analysis resulted in 889 coded text fragments from the 59 interviews: 203 quotations related to inclusive leadership behaviour and 686 quotations related to the IMPG analysis model. Five coded text fragments indicated non-positive ILB: two fragments related to the absence of ensuring justice and equity, and three coded text fragments concerned not helping staff fully contribute. Finally, quotes from the participants were translated into English to illustrate the main findings.

## Findings

### Learning logs

A total of 110 learning logs were completed by 17 participants (number of logs per participant: min = 1, max = 17, modus = 5). Out of the 110 learning logs, 92 pertained to the category of facilitating belongingness, 8 pertained to valuing uniqueness, and 10 were coded ‘other’. Three learning logs indicated non-positive ILB: one learning experience pertained to the absence of role modelling, and two experiences were related to the lack of justice and equity. First, the learning experiences are described within the ILB framework, followed by the participants’ learning pathways. Subsequently, examples are provided through quotations that describe (multiple) learning pathways, offering insights into both the discussed content related to ILB and the pursued learning pathways.

Table 3 provides an overview of the learning logs per participant concerning the ILB aspects to which their learning experiences are linked. The table illustrates that the dimensions and aspects of ILB do not occur uniformly among the participants’ learning logs. First, relatively few participants described learning experiences related to valuing uniqueness in the learning logs. The learning experiences are concentrated around ‘role model in belongingness’, ‘supports individuals as group members’ and ‘ensures justice and equity’, all of which fall into the category ‘typical’.

**Table 3.** Descriptives and overview of learning logs per participant concerning inclusive leadership behaviour.

Name child centre (fictional)	Pseudonym participant	Sector	Number of learning logs	Supports individuals as group members		Ensures justice and equity		Organises participation and information sharing		Role model in belongingness		Helps staff to fully contribute		Encourage diverse contributions		Role model for valuing uniqueness	
Aquilegia	Andrew	Education	11	x				x		x				x			
	Adeline	Childcare	8							x							
Begonia	Barbara	Education	8	x				x									
	Beatrice	Childcare	5					x		x							
Camellia	Cecilia	Education	5	x						x							
	Charlotte	Childcare	5	x						x							
Dahlia	Daphne	Education	5			x								x			
	Deborah	Childcare	8	x		x				x		x					
Edelweiss	Ellen	Education	4	x		x											
	Emily	Education	9	x		x		x		x							
	Esther	Childcare	8			x		x		x		x					
Freesia	Felicia	Education	1	x													
	Francine	Childcare	5					x		x							
Gerbera	Gaby	Education	8	x				x		x				x			
	Gwendolyn	Childcare	1	x													
Hydrangea	Hanna	Education	17	x		x		x		x							
	Hilda	Childcare	2					x		x							
Totals				11		6		9		12		2		3		0	

**Figure 4.**
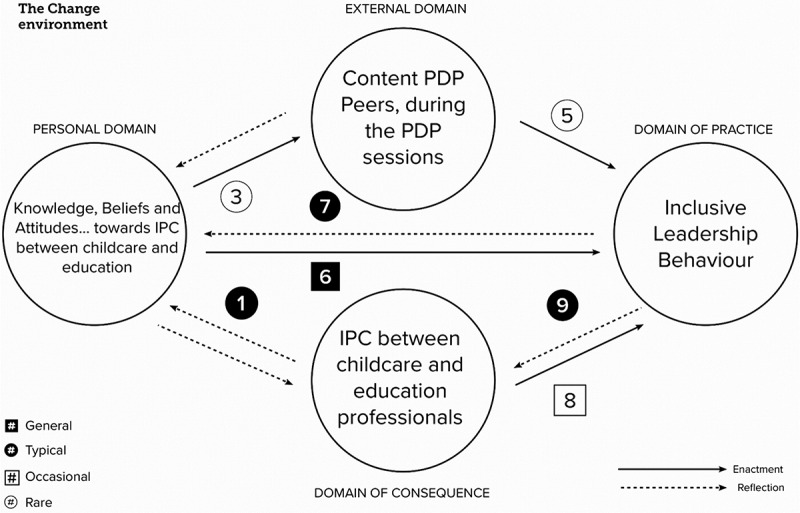
Findings for IMPG learning logs, based on the classification by Hill et al. (2005).

The second analysis focuses on learning pathways. Figure 4 illustrates that seven out of nine possible learning pathways were described in the learning logs. Of these, only one learning pathway falls under the classification ‘general’, namely: pathway 6, involving direct action in practice based on personal knowledge, beliefs, and attitudes, which, for instance, meant that participants adjusted their actions based on the knowledge they had gained. For example, participants experimented with a new ILB. Three learning pathways can be categorised as ‘typical’, meaning that more than half of the participants (but not all) followed these learning pathways. These are the learning pathways, 1. CD-PD [from consequence to personal], 7. DP-PD [from practice to personal] and 9. DP-CD [from practice to consequence], all three are based on reflection. Occasional, indicating that only one or two participants followed these learning pathways, is pathway 8. CD-DP [from consequence to practice], which involves action based on a practical situation, is rare. PD-ED [from personal to external] and 5. ED-DP [from external to practice].

The display of the learning pathway number indicates the classification.

The co-occurrence analysis, the third analysis, provided insights into the participants’ multiple learning pathways. Eight participants followed a CD-PD-DP pathway, eight participants followed a PD-DP-CD pathway, briefly reflecting on IPC as a salient outcome of ILB, and eight participants followed a PD-DP-PD pathway, reflecting on their professional experimentation.

An example of a multiple learning pathway, CD-PD-DP, related to facilitating belongingness by organising participation and knowledge sharing was demonstrated by school leader Gaby. Teachers complained to her that ‘OSC children’ did not tidy up the play materials properly (CD) [implicitly suggesting that ‘OSC-children’ did not fall under their purview, and that they also did not feel obliged to express this directly to OSC staff]. Gaby’s personal belief (PD) is that there is no such thing as ‘OSC children’, but that all children in the child centre are ‘our’ children. She communicated this to the teachers (DP) and encouraged them to directly engage in dialogue with the OSC staff to make joint agreements regarding the tidying up of materials, as they share responsibility for all children.

An example of the PD-DP-CD route, concerning facilitating belongingness by ensuring justice and equity, was illustrated by school leader Barbara, who wrote:

I believe it is important to treat everyone in the child centre equally (PD). That is why I arranged for Santa’s visit to also include the toddlers and all employees, so that both teachers and childcare workers received a small gift (DP). This gesture was highly appreciated by the staff, as I heard back.

(CD)

### Reflective interviews

This section presents the results of the reflective interviews, beginning with a description of learning experiences within the ILB framework, followed by insights into the learning pathways pursued. In addition, examples are provided to illuminate both the content of the learning experience related to ILB and the (multiple) pathways taken. Hill et al. (2005) recommended using examples to provide rich descriptions of particularly general and typical categories.

To understand the dimensions and aspects of the powerful learning experiences discussed during the reflective interviews, we refer to Table 4. The table clarifies that the number of participants describing learning experiences related to valuing uniqueness is notably lower than the number of participants describing experiences related to facilitating belongingness, although the difference between the two dimensions is noticeably smaller than in the case of the learning logs. Five aspects fall under the ‘typical’ category: ‘supports individuals as group members’, ‘ensures justice and equity’, ‘organises participation and information sharing’, ‘role model in belongingness’, and ‘helps staff to fully contribute’. It is noteworthy that the latter is an aspect of ‘valuing uniqueness’. The other two behaviours, both aspects of valuing uniqueness, fall under the ‘occasional’ category.

**Table 4.** Descriptives and overview of reflective interviews per participant concerning inclusive leadership behaviour.

Name child centre (fictional)	Pseudonym participant	Sector	Number of reflective interviews	Supports individuals as group members		Ensures justice and equity		Organises participation and information sharing		Role model in belongingness		Helps staff to fully contribute		Encourage diverse contributions		Role model for valuing uniqueness	
Aquilegia	Andrew	Education	3			x		x		x						x	
	Adeline	Childcare	4	x		x		x		x		x		x			
Begonia	Barbara	Education	3			x		x		x				x		x	
	Beatrice	Childcare	2							x		x		x			
Camellia	Cecilia	Education	4	x		x		x		x		x					
	Charlotte	Childcare	4	x		x		x		x		x				x	
Dahlia	Daphne	Education	4			x		x		x		x		x			
	Deborah	Childcare	4	x		x		x		x		x		x			
Edelweiss	Ellen	Education	3	x		x		x		x						x	
	Emily	Education	4	x		x		x		x		x				x	
	Esther	Childcare	4	x		x		x		x		x					
Freesia	Felicia	Education	3	x				x				x		x			
	Francine	Childcare	3	x				x									
Gerbera	Gaby	Education	3	x				x		x						x	
	Gwendolyn	Childcare	4	x		x				x		x				x	
Hydrangea	Hanna	Education	3	x				x		x							
	Hilda	Childcare	4											x			
Totals				12		11		14		14		10		7		7	

**Figure 5.**
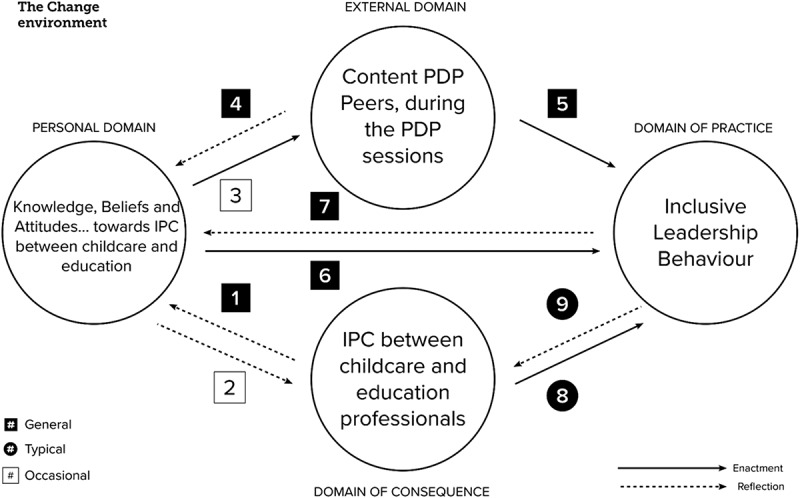
Findings for IMPG reflective interviews, based on the classification by Hill et al. (2005).

Figure 5 shows that based on all the reflective interviews, five learning pathways were classified as generally occurring among our sample: 1. CD-PD [from consequence to personal] 4. ED-PD [from external to personal], 5. ED-DP [from external to practice], 6. PD-DP [from personal to practice] and 7. DP-PD [from practice to personal]. Two learning pathways were classified as typical, 8. CD-DP [from consequence to practice] and 9. DP-CD [from practice to consequence] another two, as occasionally occurring: 2. PD-CD [from personal to consequence] and 3. PD-ED [from personal to external].

The display of the learning pathway number indicates the classification.

Similar to the learning logs, a co-occurrence analysis was then conducted to yield insights into the multiple learning pathways followed by the participants. Based on this, three examples are elaborated below, illustrating these multiple learning pathways while also providing clarity regarding the underlying single learning pathways. In all instances, these single learning pathways represent generally occurring pathways, meaning that at least 16 out of the 17 participants described a powerful learning experience based on these pathways.

*CD-PD-DP path*. This multiple learning pathway was described by nine participants. It originates from the domain of consequence, where a participant engages in reflection in response to a particular event and subsequently acts based on this introspection. Cecilia’s example clearly illustrates her understanding of community building as an aspect of facilitating belongingness, which extended to all staff members within the child centre. She shared her professional perspective with the management team colleagues.

In the management team meeting, I had a bit of a setback when a colleague mentioned that ‘getting to know each other is not that important for primary teachers in the upper grades’ (CD). However, as I have truly reflected on this matter (PD), I spoke up and said: ‘Actually it is important because they are also part of our child centre. This requires a certain mindset that all staff should embody. Also, in the upper grades, you have responsibilities in that regard. We all work in a child centre, and that holds significance for every single one of us’. (DP)

(Cecilia, reflective interview 3, 11:4)

*ED-PD-DP path*. This multiple learning pathway was also found in nine participants. Hanna’s experience is a practical example of this. In her first interview, Hanna mentioned the green keychain backpack that all participants received during the first session to remind them of the importance of PDP in their daily practice. This, together with the conversation during the session about the importance of strong relationships from a belongingness perspective, initiated a change in the personal domain, which Hanna described as follows:

I have the green backpack keychain you gave us during the session, here on my desk, to remind me (ED). And now I think about childcare workers more often, it’s a bit of awareness. (PD) And now I am actively visiting them more often for a little chat. Like asking how their weekend was or something. (DP)

(Hanna, reflective interview 1, 52:38)

*ED-DP-PD-Path*. This multiple learning pathway was identified in seven participants. In the following example, Hanna and Hilda engage in conversation following the PDP session, during which goals of child centre development are discussed. They planned a meeting to further discuss this matter.

Last week, after the session (ED), Hanna and I had a follow-up meeting (DP), primarily to discuss why we wanted to be a child centre. Are we doing this for external perception or in the best interest of the child, and what does that entail? I found this discussion precious because it was the first time Hanna and I had talked about the purpose of becoming a child centre. When I truly reflect on it, I find it particularly beneficial to facilitate transition moments. Making those moments easier for children. On the other hand, I consider it a challenge for a child to spend the entire day in the same place. In my opinion, it is also good for children to have the freedom to spend time in a different setting. (PD)

(Hilda, reflective interview 2, 54:1)

## Discussion

The aim of this study was to enrich the knowledge base on inclusive leadership by providing insights into how school and childcare leaders develop their inclusive leadership behaviours in the context of transformation. To this end, we addressed the following research question: ‘How do school and childcare leaders develop their inclusive leadership behaviours in the workplace during a dedicated professional development programme?’

The results revealed that the participants predominantly focused on fostering belongingness in their learning logs. Although this aligns with expectations set forth by Randel et al. (2018) and recent empirical findings by Verheijen-Tiemstra et al. (2024a), it is noted that an inclusive organisational climate requires both a sense of belongingness and valuing uniqueness. Reflective interviews revealed an additional awareness of developing ILB by valuing uniqueness, suggesting that guided reflection instigated the exploration of valuing uniqueness to a larger extent than did the learning logs.

The findings concerning the learning logs revealed a predominant engagement among participants in a learning pathway where the personal domain was interconnected with the domain of practice through enactment, involving direct experimentation in professional contexts. The subsequent inclusion of reflective interviews expanded the study’s understanding, exposing more extensive learning pathways that incorporated reflective practices as a general occurrence. While school leaders typically favour learning through reflection (Hulsbos et al., 2015), this can be challenging in practice (Neeleman, 2019). The broader repertoire of reflective learning pathways identified through reflective interviews is an indication of this challenge.

The multiple pathways encompass all domains, potentially indicating sustainable professional growth in alignment with Clarke and Hollingsworth’s framework. Our study highlights the efficacy of integrating learning logs with guided reflection as an intervention, as advocated by Greenan (2023). The fact that someone (in our study: the first author as a researcher and facilitator of the four sessions) posed questions aimed at eliciting deeper introspection, enabling participants to examine and articulate their learning experiences, adds value compared to independent (written) reflection on learning experiences. This aligns with Schipper et al. (2017), who highlighted the benefits of an external facilitator in the PDP process. In addition, our findings resonate with qualitative research benefits in leadership learning, as previously advocated by Brown et al. (2011).

Our study suggests heightened participant awareness in consciously capitalising on ILB opportunities, as indicated by the scarcity of non-positive ILB in our study. Verheijen-Tiemstra et al. (2024a) suggested that non-positive behaviours may result from unconscious actions, causing leaders to unintentionally miss opportunities to exhibit ILB. The current findings suggest that professional growth within the personal domain has occurred, leading to a heightened awareness of inclusive leadership, which is critical for enhancing collaboration and cohesion (Simmons & Yawson, 2022).

A strength of our study lies in its longitudinal and triangulated data collection approach. The study incorporated four interview sessions with each participant, and the learning logs were documented over a period of 5 months. This approach enabled us to demonstrate that participants exhibited varying learning pathways over time, ultimately expanding their professional repertoire throughout the duration of the PDP. Our findings align with Clarke and Hollingsworth’s (2002) conceptualisation of professional growth, which suggests that the occurrence of change is more than momentary and that changes in knowledge or beliefs are necessary to realise professional growth. Our findings suggest that the combination of offering thoughtfully selected content and employing learning logs and reflective interviews affords numerous learning opportunities, thereby contributing to perceived professional growth.

Our study also has some limitations. A constraint of our study pertains to the methodological limitation that, for practical consideration, we exclusively relied on data gathered through interviews and learning logs, without supplementing our data with observational data of actual behaviours. The second limitation concerns the overlap between the learning experiences described in the learning logs and reflective interviews. On the one hand, not every respondent completed one or more learning experiences for each interview, while on the other hand, some respondents had described several learning experiences in the learning logs, which required a choice to be made during the interview regarding which experience the participant considered most valuable to discuss. This may be an outgrowth of the decision not to make the writing of learning logs mandatory. In line with (Greenan, 2023), time and/or work pressure could be barriers to completing learning logs. Another possible explanation is that learning logs had to be uploaded to a distinct learning environment. For future research we propose implementing practical alternatives, such as enabling the completion of learning logs via a mobile application.

### Implications for theory and practice

From a theoretical perspective, this study advances the understanding of inclusive leadership by integrating it into the IMPG framework developed by Clarke and Hollingsworth (2002). This combination adds to the existing HRD literature by providing a detailed account of how leaders’ learning processes are structured and operationalised in real-time leadership scenarios. Moreover, the study strengthens the current debate on leadership and culture by articulating the interaction between leader development and national cultural norms in a low-power distance culture like the Netherlands. National culture may influence how leaders perceive inclusion and the distance, if any, between inclusive leadership behaviours such as information sharing and participation in decision making and the leaders’ own norms, values and beliefs following from this culture. Consequently, the findings of this study may not be readily generalisable to national cultures that are markedly different in terms of cultural values, social structures, and individualistic versus collective orientations. However, our study provides a framework for investigating this profound insight by integrating IMPB and inclusive leadership into diverse national cultural contexts.

The combination of a PDP with learning logs and reflective interviews revealed a more comprehensive range of learning pathways, thus shedding more light on the learning processes of the leaders who participated in this study. As our study shows, participants create their own unique learning pathways that are tailored to their individual needs and contextual factors that they consider important. This is the first possible explanation for the different outcomes that participants derive from participation in the PDP. Additionally, it indicates that while learning logs show a generally occurring direct learning pathway from the personal domain to the domain of practice, guided reflection led to other generally occurring indirect learning pathways, such as those from the external domain to the personal domain, where the external domain led to reflection on participants’ beliefs or attitude. This indicates that guided reflection may be an effective method for achieving in-depth reflection because the probing questions during the reflective interviews facilitate a more comprehensive examination of the reflection steps between the domain of practice and the personal domain.

A suggestion for future HRD research regarding the use of the IMPG would be to conduct an in-depth case study to delve into the process of reflection in workplace learning in greater detail to study whether different forms of reflection occur in different domains and learning pathways. Clarke and Hollingsworth (2002) did not offer a detailed operationalisation of the concept of ‘reflection’, and Justice et al. (2020) advocate for more research aiming to operationalise reflection as an activity embedded in workplace practices.

Future HRD research on this topic should focus on different contexts, such as geographical boundaries, different institutional logics, and organisational cultures. It may be relevant to study how ILB behaviours may vary in contexts characterised by higher power distance or more collectively oriented national cultures and how this may affect learners’ learning pathways in ILB. The methods used in our study offer possibilities for such comparisons between countries and/or sectors, including the public and private sector (including SMEs and corporate organisations). This would enable a signal of whether differences in the focus on belongingness and uniqueness are more widely recognised, as well as the investigation of whether there is a shift over time.

Regarding implications for practice, HRD professionals could focus on organising and supporting learning activities based on this PDP and on inclusive leadership in contexts where IPC is considered increasingly important. It is recommended to make sufficient time and space available in such learning activities for facilitating guided reflection in addition to written reflections as part of the PDP, aiming at awareness of relational processes among employees with diverse backgrounds.
